# Caseload, treatment diagnoses, and evaluation of clinical care concepts for infectious diseases at a municipal tertiary care hospital in Germany

**DOI:** 10.1007/s15010-026-02902-y

**Published:** 2026-07-31

**Authors:** Kathrin Marx, Nils Kellner, Nadja Herrmann, Christoph Lübbert

**Affiliations:** 1 Hospital Pharmacy, Hospital St. Georg, Leipzig, Germany; 2 Department of Infectious Diseases and Tropical Medicine, Hospital St. Georg, Leipzig, Germany; 3 Department of Finance and Controlling, Hospital St. Georg, Leipzig, Germany; 4 Division of Infectious Diseases and Tropical Medicine, Department of Medicine I, Leipzig University Medical Center, Liebigstr. 20, D-04103 Leipzig, Germany; 5 Interdisciplinary Center for Infectious Diseases, Leipzig University Medical Center, Leipzig, Germany

**Keywords:** Hospitalization, ICD-10 diagnoses, Specialization, Antimicrobial stewardship (AMS), Antibiotic stewardship (ABS), Infectious disease consultation service, Costs, Reimbursement, G-DRG system, Economic analysis

## Abstract

**Background:**

Comprehensive data on municipal specialized infectious disease (ID) departments in Germany are scarce.

**Purpose:**

To analyze ID cases at Hospital St. Georg in Leipzig with regard to patient characteristics, spectrum of infections, care concepts, length of stay (LOS), clinical outcomes, and economic indicators.

**Methods:**

Retrospective descriptive analysis of all adult inpatient cases between January 2020 and December 2025.

**Results:**

Of 17,878 adult patients (11.5% of all patients) discharged with a primary ID diagnosis, 28.7% (5,135/17,878) were treated as inpatients in the 40-bed ID department itself, while 3.8% (671/17,878) received organ-specific treatment in another specialty department with concurrent care provided by the antimicrobial stewardship (AMS) team, and 5.2% (935/17,878) received care via the ID consultation service (telephone consultations not included). A total of 4,492 individual ICD-10 codes were assigned, with 740 of them being classified as ID diagnoses. ID patients were older (median 70 vs. 66 years) and more frequently male (59% vs. 51%) than the reference population. The median LOS was longer for ID patients (7.0 vs. 4.1 days). The median case-mix index (CMI) was 1.344 (€ 5,356 per case) in the ID population vs. 1.113 (€ 4,352 per case) in the reference group.

**Conclusions:**

This six-year analysis shows that ID patients represent a significant, complex, and older inpatient population with longer LOS, causing high clinical burden. Since coding data suggest that 62.3% of these patients did not receive specific treatment by ID specialists, there is potential for expanding structured ID consultation and AMS services, requiring additional resources.

**Supplementary Information:**

The online version contains supplementary material available at https://doi.org/10.1007/s15010-026-02902-y.

## Introduction

Infectious diseases (IDs) present substantial medical, organizational, and socio-economic challenges within the German healthcare system. Beyond their direct impact on patient morbidity and mortality, IDs exert profound structural and financial pressures on healthcare infrastructure [1, 2]. Addressing these challenges necessitates a multi-faceted, interdisciplinary approach that integrates epidemiology. clinical management, stewardship, hygiene protocols, healthcare structure, and hospital economics.

The long-held assumption that IDs could be permanently controlled through the widespread use of anti-infectives, particularly antibiotics, proved to be erroneous many years ago [3]. The inappropriate and unregulated use of these substances has significantly accelerated the emergence of resistant pathogens and thus increasingly limited anti-infective treatment options [4–6]. This global crisis demands continuous innovations in diagnostics and therapeutics, alongside a systematic expansion of ID expertise across both outpatient and inpatient sectors [7]. Despite the high complexity and resource intensity of specialized ID management, the German diagnosis-related groups (G-DRG) system fails to adequately account for ID expertise or the economic value of specialized care infrastructure, such as ID outpatient clinics, antimicrobial stewardship (AMS) programs, and structured ID consultations. Consequently, these highly individualized, resource-intensive cases are poorly represented by a flat-rate reimbursement system based on average costs, highlighting a critical structural deficit in current German healthcare financing [8]. In contrast, particularly in the field of IDs, the G-DRG system creates strong economic incentives that are not always compatible with high-quality, complex, or specialized care. Tightening fiscal constraints often exert substantial pressure on hospitals to shorten the length of stay (LOS), potentially compromising clinical outcomes. While the current framework rewards standardized, predictable clinical pathways, it penalizes complex, patient-centered interventions that exceed standard resource allocations, thereby generating financial deficits. Under this model, medical complexity is primarily captured through secondary diagnoses and procedural codes, failing to reflect the specialized expertise, professional competencies, or structural infrastructure of the treating ID team [2, 9].

To address these systemic challenges, this study aims to quantify the prevalence of ID-related patient care at a German municipal tertiary care hospital equipped with a large, independent, and specialized ID department. Furthermore, we systematically evaluate patient demographics, the spectrum of infections, therapeutic strategies, LOS, clinical outcomes, and economic indicators to identify structural inefficiencies and areas for optimization.

## Methods

### Study design, setting and patients

This single-center, retrospective cohort study was conducted at Hospital St. Georg, a municipal tertiary care center in Germany with over 700 beds spanning 25 specialized departments and clinics. The study period encompassed admissions between January 1, 2020, and December 31, 2025. All patients aged ≥ 18 years were eligible for inclusion. Hospital St. Georg is characterized by a specialized, high-acuity infrastructure, featuring large departments for severe burns, septic surgery, and an independent 40-bed ID department, which includes a dedicated high-level isolation unit for highly pathogenic infectious agents.

### ICD-10 diagnoses

ID cases were identified based on the primary ICD-10 discharge diagnosis. Patients matching these criteria were assigned to the ID cohort, while all remaining patients served as the reference population. To facilitate detailed analysis, primary ID-related ICD-10 codes were categorized into the following mutually exclusive infection groups:

1. Infections of the upper respiratory tract: tonsillitis, sinusitis, infectious mononucleosis, pharyngitis, laryngitis, tracheitis (ICD-10 codes: B00, B08, B27, J00-J06, J36, J39).
2. Infections of the lower respiratory tract: bronchitis, bronchiolitis, exacerbation of COPD triggered by infection; infections caused by influenza and parainfluenza viruses, RSV, human metapneumovirus, or *Bordetella pertussis*; lung abscess, infections of the mediastinum and the pleural space (ICD-10 codes: A37.0, J09-J11, J20-J22, J44.0, J85-J86).
3. Radiologically confirmed pneumonia (ICD-10 codes: A48.1, B05.2, B25.0, J10.0, J11.0, J12-J18, J69, J85.1).
4. Coronavirus disease 2019 (Covid-19) (ICD-10 code: B34.2, in conjunction with the confirmatory ICD-10 code U07.1).
5. Infections of the central nervous system (CNS): meningitis, encephalitis, myelitis, brain abscess (ICD-10 codes: A32.1, A39.0, A81, A84-A87, B00.3, B00.4, B01.0, B01.1, B02.0, B02.1, B58.2, B69.0, G00, G03-G04, G06.0).
6. Infections of the gastrointestinal tract and the abdominal cavity: esophagitis, gastroenteritis, enteritis, colitis, enterocolitis, diverticulitis, appendicitis, anal/rectal abscess, cholecystitis, cholangitis, liver abscess, hepatitis (acute and chronic), peritonitis, intraabdominal abscess (ICD-10 codes: A01-A04, A06-A09, A54.6, B15—B19, B25.1, B25.8, K20, K29, K35, K57.2, K57.3, K57.4, K57.8, K61, K63, K65, K75, K80-K81, K83).
7. Infections of the urinary tract: cystitis, pyelonephritis, interstitial nephritis, renal abscess, perinephric abscess, infections of the urethra, infections of the prostate gland (ICD-10 codes: A54.0, A60.0, N10-N12, N13.6, N15.1, N30, N34, N39.0, N41, N45, O23).
8. Infections of the skin and soft tissues: erysipelas, cellulitis, fasciitis, tendovaginitis, lymphangitis, wound infections (ICD-10 codes: A21, A31, A36.3, A46, A51, A55, A69, B00- B02, B04, B08-B09, B86-B87, H60, J34.0, K12, L00-L05, L08, M65, M71.0, M71.1, M72.6, O86, O91, T79-T80, T89.02).
9. Infections of bones, joints, and spine: arthritis, osteomyelitis, osteitis, spondylarthritis, spondylodiscitis, discitis, intraspinal, extradural, subdural abscesses (ICD-10 codes: G06.1, M00, M02, M46, M86).
10. Infections associated with prostheses, implants, transplants, grafts and other foreign bodies, including prosthetic joint infections and infections related to implants for osteosynthesis (ICD-10 codes: T82.6, T82.7, T83.5, T83.6, T84.5, T84.6, T84.7, T85.7).
11. Infections of the heart: endocarditis, myocarditis, pericarditis (ICD-10 codes: I30.1, I32, I33.0, I39, I40.0, I41).
12. Sepsis, septic shock, toxic shock syndrome (ICD-10 codes: A02.1, A32.7, A39.2, A40-A41, A48.3, B37.7, R65.1).
13. Tuberculosis (TB) and other mycobacterioses (ICD-10 codes: A15-A16, A18-A19, A31).
14. HIV-related infections and immune reconstitution inflammatory syndrome (IRIS), excluding pneumocystosis, toxoplasma encephalitis, cryptococcosis, and other opportunistic diseases coded elsewhere (ICD-10 codes: B20.1-B20.9, B21-B24).
15. Protozoan infections: malaria, leishmaniasis, toxoplasmosis (ICD-10 codes: B50-B55, B58).
16. Fungal infections: aspergillosis, candidiasis, histoplasmosis, mycetoma, cryptococcosis, dermatophyte infections, coccidioidomycosis, zygomycosis (ICD-10 codes: B35, B37-B39, B44-B48).

### Data collection and data processing

The study utilized all data routinely recorded through the hospital information system. After extraction, the data were analyzed anonymously. The collected information included patients’ age at admission, LOS, DRG-related case mix points (CMPs), and in-hospital mortality. The case mix index (CMI) was calculated by dividing the sum of the CMPs by the total number of cases. The CMI reflects the weighting of DRG reimbursement, but only indirectly captures aspects of severity and resource consumption. Revenue per case was calculated by multiplying the CMI by the national base rate for the Free State of Saxony [10]. Additionally, information on the LOS deviation was collected, resulting from the difference between the actual LOS and the number of bed occupancy days used to calculate the DRG flat rate. In the G-DRG framework, the term “inliers” refers to cases whose LOS falls within the expected range of LOS, which is derived from the majority of cases within a specific DRG. For inliers, hospital costs and reimbursements are largely in line with one another. In contrast, outliers represent patient cases that deviate from the expected LOS within a DRG. Outliers are divided into low and high outliers, depending on whether they fall below the lower or above the upper DRG limit. High outliers therefore place a burden on hospital budgets, while low outliers can increase hospital profits [2].

### Statistical analysis

Descriptive analyses were performed to compare the ID population with the reference population. Median and interquartile range (IQR) were calculated for continuous variables, and absolute and relative frequencies for categorical variables. The odds ratio (OR) for in-hospital mortality in the ID population compared to the reference group was determined using logistic regression, taking age at admission, sex, treatment in an intensive care unit (ICU), and surgical procedures into account. Quantitative variables were compared using the Mann-Whitney U test, qualitative variables were compared using the Chi-square test or Fisher’s exact test, as appropriate. A p-value < 0.05 was considered statistically significant. All analyses were performed using the statistical software R (version 4.5.2).

### Ethical considerations

This study used aggregated and anonymized data and therefore did not require approval from institutional review boards or ethical committees or informed consent from patients. The study was conducted in accordance with the ethical guidelines of the 1964 Declaration of Helsinki and its later amendments.

## Results

### Demographic characteristics of patients and clinical spectrum of infectious diseases

During the study period (2020–2025), 155,299 adult patients received inpatient treatment (baseline population). A total of 4,492 individual ICD-10 codes were assigned to these patients. Patients with primary diagnoses classified as IDs were assigned 740 different ICD-10 codes.

A total of 17,878 patients were discharged with a primary ID diagnosis, representing 11.5% of all patients. The reference population consisted of the remaining 137,421 cases without a primary ID diagnosis. The case characteristics of the ID and reference populations are summarized in Table 1. The number of ID cases per ICD-10 code ranged from 2,025 (N39.0, urinary tract infection) to 1 (T85.75, infection and inflammatory reaction due to internal prostheses, implants, or transplants in the hepatobiliary system and pancreas) (Supplementary Table 1). The twelve and the 87 most frequently used ICD-10 codes accounted for approximately 40% and 80% of all ID cases, respectively.

In the ID population, patients were older (median 70 years vs. 66 years) and more frequently male (59% vs. 51%) than in the reference population. The median LOS for ID patients was 7.0 days, compared to 4.1 days in the reference population. Similarly, the inliers and high outliers were 4.6% and 3.8% higher, respectively, than the corresponding values in the reference population. 28.7% (5,135/17,878) of the ID population were treated as inpatients in the 40-bed ID department itself, while 3.8% (671/17,878) of the remaining patients received organ-specific treatment in another specialty department, with concomitant care provided by the AMS team; 5.2% (935/17,878) were co-handled by the ID consultation service, with telephone consultations not included.

**Table 1:** Case characteristics of the study population

	ID population	Reference population
Cases (*n*) (%)	17,878 (11.5)	137,421 (88.5)
Female	7,333 (41.0)	67,699 (49.3)
Male	10,545 (59.0)	69,721 (50.7)
Diverse	0 (0.0)	1 (0.0)
Age (years) (IQR)	70.0 (54.0, 81.0)	66.0 (48.0, 78.0)
18–49	3,704 (20.7)	35,553 (25.9)
50–79	8,674 (48.5)	70,708 (51.5)
≥ 80	5,500 (30.8)	31,160 (22.7)
Length of stay (days) (IQR)	7.0 (4.0, 11.7)	4.1 (2.1, 8.0)
Low outliers	1,451 (8.3)	22,789 (16.6)
Inliers	14,024 (80.5)	103,986 (75.9)
High outliers	1,952 (11.2)	10,150 (7.4)
Deviation in length of stay	0.0 (-2.0, 3.0)	-1.0 [-2.0, 1.0]
Recurring cases (n) (%)	413 (2.3)	3,496 (2.5)
In-hospital mortality (n) (%)	1,446 (8.1)	4,442 (3.2)
Cases treated in the ID department (n) (%)	5,135 (28.7)	2,159 (1.6)
Cases with ICU treatment (n) (%)	1,975 (11.0)	11,816 (8.6)
Cases seen by the ID consultation service (n) (%)	935 (5.2)	1,950 (1.4)
Cases with concurrent care by the AMS team (n) (%)	671 (3.8)	1,053 (0.8)

AMS, antimicrobial stewardship; ICU, intensive care unit; ID, infectious disease; IQR, interquartile range. Data are presented as numbers (percentages) or median (IQR: Q1, Q3)

The proportion of mutually exclusive infection groups in the total ID population is summarized in Supplementary Table 2. The most common infection was pneumonia (19.8%), followed by urinary tract infections (15.5%), and skin and soft tissue infections (11.9%). 4.1% of patients could not be classified into given categories and were therefore summarized as “other infections”.

### Economic impact

The distribution of the CMI and the revenue volume of the cases is summarized in Supplementary Table 3. The median CMI was 1.344 (IQR 1.290–1.421) in the ID population, corresponding to € 5,356 per case, compared with 1.113 (IQR 1.088–1.150) in the reference population, corresponding to € 4,352 per case. The median CMI for cases in the ID population treated in the ID department itself was 1.281 (IQR 1.207–1.324), compared with 1.389 (IQR 1.325–1.471) for the remaining ID patients treated in another specialty department. The revenue volume was lower for cases with a stay in the ID department (€ 4,956) than for cases with a stay in another specialty department (€ 5,469).

**Fig. 1:**
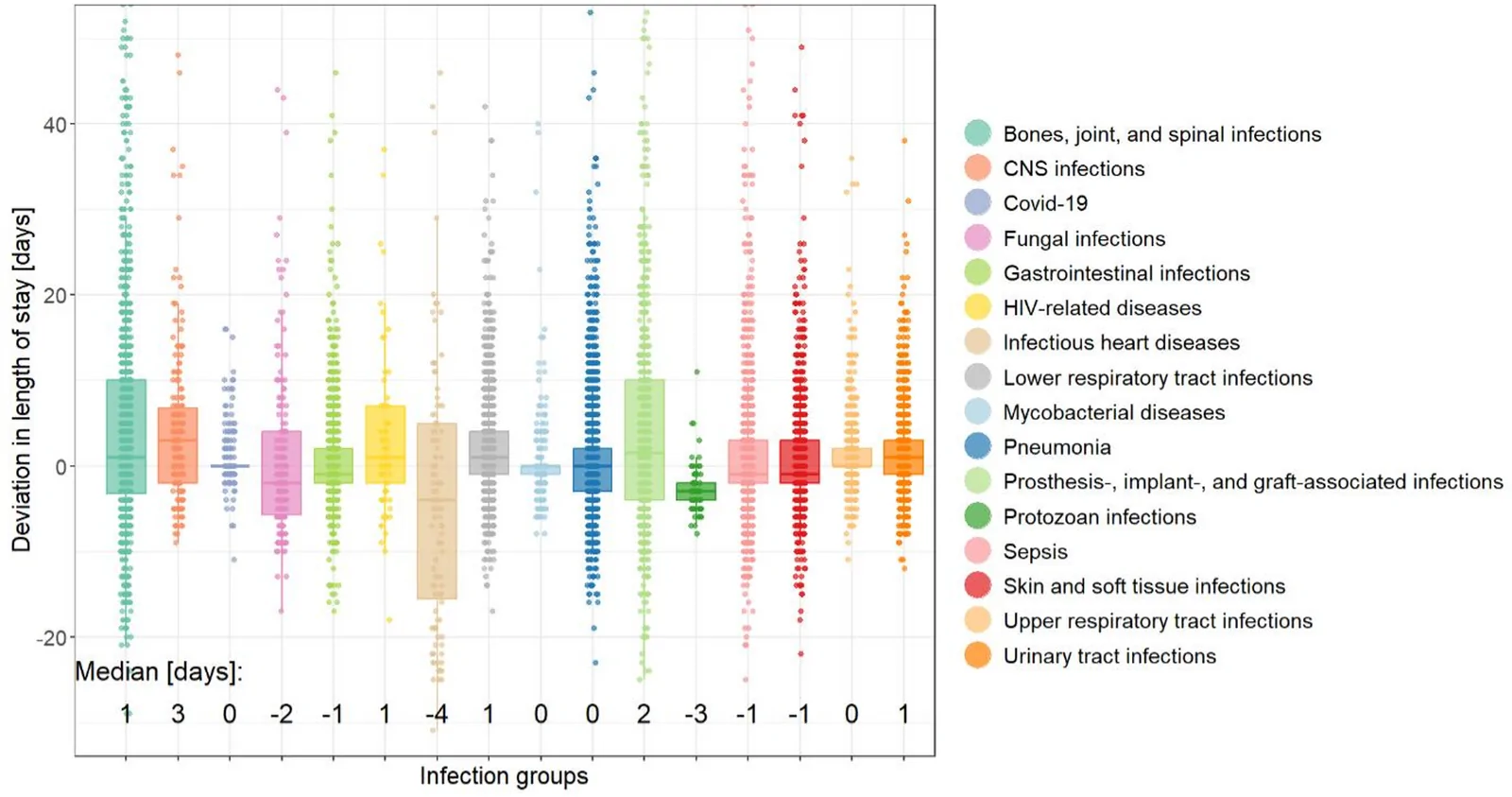
Length of stay and median deviation from the DRG catalog in days, stratified by infection groups

We also collected data on the deviation in LOS — that is, the difference between the actual LOS and the number of bed-days used to calculate the DRG flat rate. The median deviation in LOS, stratified by infection group, is summarized in Supplementary Tables 4 and presented in Fig. 1. With the exception of the infection groups pneumonia, upper respiratory tract infections, Covid-19, and mycobacterial diseases — for which the actual average LOS did not deviate from that calculated in the DRG catalog — reductions in DRG reimbursements are to be expected due to deviations from the DRG-specific LOS.

### Age distribution and in-hospital mortality associated with infectious diseases

**Fig. 2:**
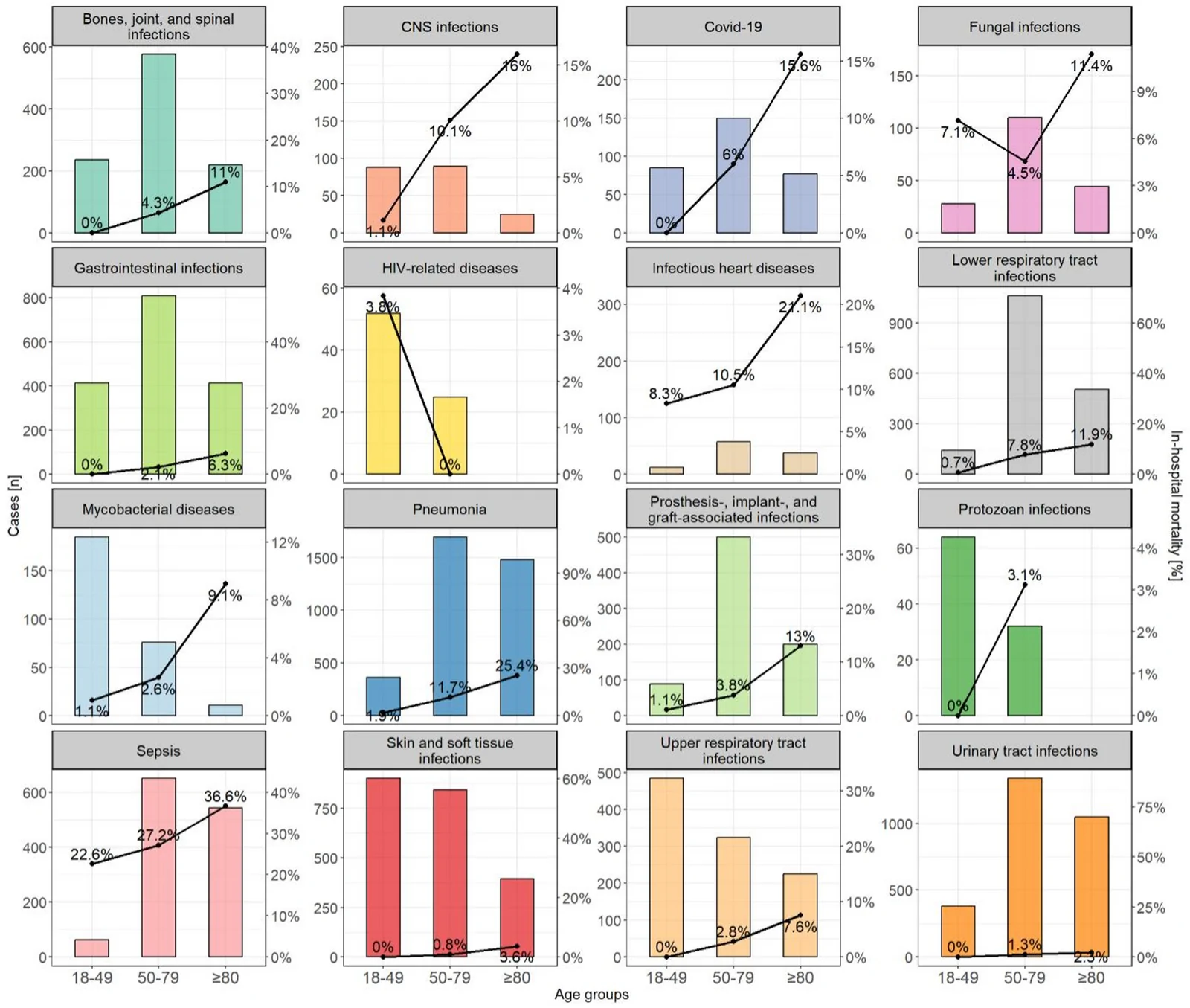
Presentation of the age distribution, divided into different age groups (columns with percentages), with the corresponding in-hospital mortality rates (black lines), stratified by infection groups

The in-hospital mortality rate was significantly higher in the ID population (8.1%) than in the reference population (3.2%). It was age-dependent across all infection groups, with the exception of HIV-associated conditions, and was highest among patients aged ≥ 80 years with sepsis, reaching 36.6% in this group. Urinary tract infections were particularly common in this age group (1,048 cases; 19.1%), but showed the lowest mortality rate (2.3%). CNS infections were relatively rare in the age group ≥ 80 years (25 cases, 0.5%), but with a high mortality rate of 16.0%. All results are presented graphically in Fig. 2. Additional information is given in Supplementary Table 2.

**Fig. 3:**
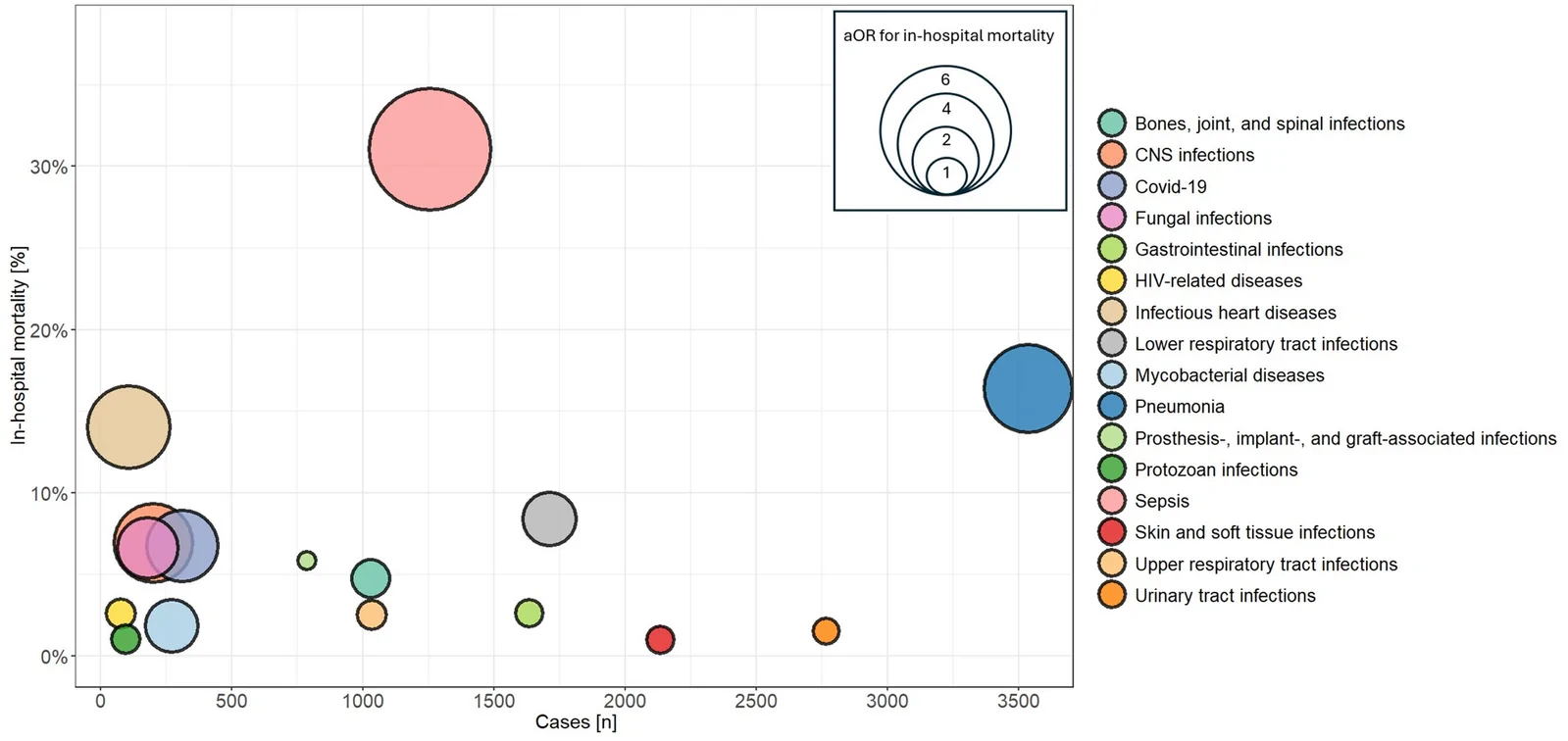
Caseload and in-hospital mortality in the ID population. The bubble chart shows infection groups plotted against the total number of infections (x-axis) and the crude in-hospital mortality rate (y-axis). The size of the bubbles is proportional to the adjusted odds ratio (aOR) for in-hospital mortality for each infection group. The color coding represents the respective infection group. Please note that aORs are not adjusted for clinical severity or comorbidities

By comparing infection groups in terms of their overall incidence, associated in-hospital mortality, and adjusted risk of in-hospital mortality, we were able to identify those that have a disproportionately large impact on the ID population. Using a logistic regression model adjusted for age at admission, sex, ICU treatment, and surgical procedures, we assessed the adjusted odds ratio (aOR) for in-hospital mortality for each infection group compared to patients without IDs (Fig. 3 and Supplementary Table 5). Notably, the mortality rate (16.4%) and the risk of in-hospital mortality (aOR 3.1, 95% confidence interval [CI] 2.8 to 3.5) for pneumonia was comparable to those for infectious heart diseases (14%) (aOR 2.8; 95% CI 1.5 to 4.9), while lower respiratory tract infections and Covid-19 (8.4% and 6.7%, respectively) also had comparable mortality rates and outcomes.

As expected, sepsis patients represented another outlier group, with a significantly higher adjusted risk of in-hospital mortality (aOR 5.8, 95% CI 5.0 to 6.6) than in other infection groups with a similar incidence of infection.

## Discussion

This study highlights the ongoing significant clinical burden posed by IDs among inpatients in Germany, with a particular focus on care aspects at one of the few specialized municipal hospitals with its own ID department. Overall, 11.5% of all hospital stays were attributable to a primary diagnosis of ID, of which 28.7% were treated as inpatients directly in the 40-bed ID department. Due to the scarcity of well-established inpatient ID structures in German municipal hospitals, these data provide novel perspectives compared to prior cross-sectional studies.

Consistent with previous reports from other healthcare settings, patients discharged with a primary ID diagnosis were characterized by advanced age, longer hospital stays, and substantially higher case complexity and reimbursement levels compared with the overall inpatient population [2]. These findings underscore that IDs represent a major component of contemporary hospital medicine and are associated with a considerable clinical burden with corresponding economic challenges. Notably, only 37.7% of patients discharged with a primary ID diagnosis received care within dedicated ID structures, either through admission to the ID department, co-management by the AMS team, or formal ID consultation. Consequently, a substantial proportion of patients with IDs (62.3% according to the coding data) were managed without direct involvement of ID specialists, highlighting considerable potential to expand specialist services [11, 12]. Several factors may explain this finding. First, the ID consultation service may be underutilized because treating physicians do not consistently seek specialist input, potentially reflecting an overestimation of their own expertise in managing IDs within organ-specific specialties. Second, limited personnel resources and the capacity of the AMS team may constrain consultation availability, particularly in a tertiary care hospital managing a high volume of patients with complex infections. Finally, underfunding and shortages of ID specialists, which have been reported across many healthcare systems, may further restrict timely access to specialist care.

The observed age distribution is consistent with demographic developments across Germany and other European countries. Increasing life expectancy, the growing prevalence of chronic diseases and cancer, widespread use of implanted medical devices, and expanding indications for iatrogenic immunosuppressive therapies have created a patient population that is increasingly susceptible to severe and complex infections [1, 2, 9]. Simultaneously, antimicrobial resistance (AMR) continues to rise and further complicates management strategies [5]. These developments underscore the increasing importance of specialized ID expertise within hospital care [13]. Previous analyses have similarly demonstrated a substantial and growing burden of IDs in German hospitals, particularly among elderly patients with multiple comorbidities [2].

The spectrum of IDs observed in our cohort reflects the epidemiology typically encountered in acute care hospitals. Respiratory tract infections, urinary tract infections, skin and soft tissue infections, bloodstream infections, and device-associated infections account for a large proportion of admissions. This distribution is consistent with previous reports from Germany and internationally. For example, Katchanov et al. demonstrated that in a municipal healthcare setting lower respiratory tract infections, urinary tract infections, and skin and soft tissue infections constitute a substantial proportion of ID-related hospitalizations and contribute significantly to inpatient resource utilization [8]. At the population level, IDs remain among the leading causes of morbidity and healthcare utilization. According to the burden of communicable diseases analysis for the European Union (EU), influenza accounts for approximately 30% of the total IDs burden, followed by tuberculosis, HIV/AIDS, and invasive pneumococcal disease [1]. Together, these findings illustrate the broad spectrum and substantial public health relevance of IDs.

An important observation of the present study is the discrepancy between evidence-based treatment recommendations and the economic framework imposed by the German diagnosis-related group (G-DRG) system. Many severe IDs require prolonged antimicrobial therapy, extensive diagnostic work-up, source control procedures, and close clinical monitoring. For example, infective endocarditis, vertebral osteomyelitis, complicated *Staphylococcus (S.) aureus* bacteremia, and invasive fungal infections frequently require treatment durations extending well beyond the average DRG-calculated length of stay [14, 15]. Consequently, hospitals face a structural mismatch between guideline-concordant care and reimbursement mechanisms. This challenge is further aggravated by the inherently unpredictable nature of IDs. Unlike many elective medical or surgical admissions, infections often occur unexpectedly, and their clinical course, complications, and LOS are difficult to anticipate. Therefore, hospital capacity planning for IDs cannot rely solely on conventional elective admission models.

Dedicated ID departments may help address this challenge by providing specialized “infection phases” of care. Such structures allow patients to receive focused diagnostic and therapeutic management during the acute phase of infection before being transferred back to organ-specific services or outpatient care pathways [13]. This model may improve resource allocation while maintaining high-quality care. However, in this context, our own data also shows that a more carefully selected allocation of ID patients to the ID department is necessary in order to improve the department-specific economic baseline data. Successful implementation requires close interdisciplinary cooperation. IDs frequently affect patients with complex underlying cardiac, surgical, oncological, nephrological, or intensive care needs. Consequently, collegial collaboration between ID specialists and other disciplines remains essential for optimal patient outcomes. The relatively low proportion of patients (3.8%) in our cohort who received AMS-supported clinical co-management highlights the potential for expansion and the importance of interdisciplinary treatment concepts.

Our findings also have implications for hospital financing. The median CMI of 1.344 among ID patients exceeded that of the reference population, indicating substantially higher resource consumption. Previous studies have demonstrated that patients requiring ID consultation are characterized by higher clinical complexity levels, longer hospital stays, and higher CMIs than patients managed without specialist involvement [2, 16]. Meyer-Schwickerath et al. showed that patients receiving ID consultation in German university hospitals were significantly more complex across multiple healthcare indicators, emphasizing the resource-intensive nature of this patient population [17]. At the same time, reimbursement structures may not adequately reflect the actual workload associated with ID care. This issue is particularly relevant for dedicated ID departments in municipal hospitals, where economic pressures are often greater than in university hospitals. Although direct comparisons remain limited, university-based ID services may benefit from tertiary referral patterns, higher case complexity, and additional academic funding streams. In contrast, municipal hospitals must frequently provide comparable expertise with fewer structural resources. Therefore, improved case selection and strategic admission management may become increasingly important for dedicated ID departments to ensure both clinical effectiveness and financial sustainability.

The limited penetration of ID consultation services observed in our cohort deserves particular attention. A growing body of evidence demonstrates that involvement of ID specialists improves patient outcomes in a variety of clinical settings [19–26] and contributes to shorter hospital stays and lower readmission rates [27]. Although randomized controlled trials remain scarce, observational studies consistently show benefits with regard to diagnostic accuracy, antimicrobial optimization, adherence to guidelines, and mortality. Particularly strong evidence exists for difficult-to-treat infections such as *S. aureus* bacteremia [14, 20, 21], candidemia [15, 22], septic shock [23], gram-negative bloodstream infections [24], infections caused by multidrug-resistant organisms [25], and infections in solid organ transplant recipients [26]. Furthermore, several studies have shown improved outcomes among patients with *S. aureus* bacteremia following formal ID consultation, including reductions in mortality and improved adherence to quality-of-care indicators [14, 20, 21, 28]. The importance of ID consultation services for German hospitals has been emphasized repeatedly, including by Rieg and Küpper, who highlighted their role in improving clinical outcomes and advocated for a broader implementation of ID expertise nationwide [19]. Recent German data further support the strategic expansion of ID consultation services. Meyer-Schwickerath et al. demonstrated that patients receiving ID consultation represent a particularly complex subgroup, suggesting that specialist involvement is directed toward patients with the greatest potential benefit [17]. Similarly, a recent European consensus paper by Hornuss et al. highlighted the growing relevance of structured ID services and multidisciplinary infection management in modern hospital care [18]. These findings collectively support further expansion of ID consultation and AMS programs. However, such expansion can only be achieved if accompanied by appropriate staffing levels and dedicated financial resources. Otherwise, increasing demand may further strain already limited specialist capacities.

Another important aspect concerns the future role of sepsis within hospital reimbursement and quality management systems. In Germany, structured sepsis quality management programs have recently been implemented, which may influence diagnostic coding practices, case identification, and resource allocation [29]. It remains to be seen how these initiatives will affect the reported incidence and economic representation of sepsis cases in future analyses. Given the close overlap between sepsis management and ID expertise, ID departments are likely to play an increasingly important role in these quality improvement efforts.

### Limitations

A major limitation of this study is its reliance on routinely collected administrative data without consistent clinical case-by-case validation. Consequently, the study quantifies the burden of administratively coded primary ID discharge diagnoses rather than the true clinical burden of IDs. ICD-10 and DRG coding are primarily designed for administrative and reimbursement purposes and may therefore be influenced by documentation quality, local coding practices, reimbursement incentives, and temporal changes in coding behavior. As a result, misclassification and incomplete case ascertainment cannot be excluded and should be considered when interpreting the findings.

Furthermore, the restriction to primary discharge diagnoses likely underestimates the overall burden of IDs in hospitalized patients. Many clinically relevant infections occur as secondary diagnoses, including hospital-acquired infections, device-associated infections, postoperative infections, ICU-associated infections, or infections complicating the course of patients admitted for non-infectious primary conditions. These patients were not included in the ID cohort despite often requiring substantial diagnostic, therapeutic, and ID specialist resources. Accordingly, the non-ID reference cohort should not be interpreted as representing patients without infections. Rather, it likely included a considerable proportion of patients with clinically relevant secondary ID diagnoses that were not captured by the study definition. This overlap may have attenuated observed differences between the cohorts and suggests that the overall hospital-wide burden attributable to IDs is likely higher than estimated in the present analysis. In addition, coding accuracy may vary between hospitals because of differences in clinical documentation, coding expertise, and institutional coding practices. Previous studies have demonstrated substantial under-coding and inter-hospital variability for sepsis coding, and similar although potentially less pronounced limitations may exist for other infectious syndromes [30]. Therefore, caution is warranted when directly comparing administrative estimates across institutions or extrapolating the present findings to other healthcare settings.

It should also be noted that the monocentric study design inherently limits the study’s representativeness. Since the study period spanned the Covid-19 pandemic and the subsequent years, temporal shifts in hospital admission patterns and coding practices may also have confounded the data. Finally, the institutional characteristics of the Hospital St. Georg in Leipzig – specifically its highly specialized infrastructure, including a dedicated 40-bed ID department and advanced high-acuity services – further limit the external validity of these findings to other municipal hospitals lacking comparable specialized resources.

## Conclusions

Despite the limitations mentioned, routinely collected administrative data provide valuable insights into healthcare utilization and economic burden at the hospital level. Because coding directly determines resource allocation and reimbursement within the G-DRG system, analyses based on administrative data remain highly relevant for hospital management and health services research. Nevertheless, the present results should be interpreted as conservative estimates of the burden associated with administratively coded primary ID diagnoses rather than comprehensive estimates of all clinically relevant infections treated in hospital. Overall, our findings reinforce previous calls for stronger institutional support of ID care as an independent clinical discipline in Germany [11, 12]. The growing burden of IDs, increasing AMR, demographic changes, and the demonstrated benefits of specialist involvement argue strongly for expanding ID departments, ID consultation services, and AMS programs. However, sustainable implementation requires reimbursement models that adequately reflect the complexity and resource consumption of ID care. Without improved funding and structural support, the capacity of German hospitals to meet future ID challenges may remain limited.

## Electronic Supplementary Material

Below is the link to the electronic supplementary material.

15010_2026_2902_MOESM1_ESM.docx

## Data Availability

No datasets were generated or analysed during the current study.
