# Supplementary material for: Caseload, treatment diagnoses, and evaluation of clinical care concepts for infectious diseases at a municipal tertiary care hospital in Germany
Source: Infection. 2026 Jul 31;54(5):2671–80. doi: 10.1007/s15010-026-02902-y (PMC13727860; doi:10.1007/s15010-026-02902-y)
Supplement: Supplementary file 1 [file 15010_2026_2902_MOESM1_ESM.docx]

**Supplementary Tables**

***Supplementary Table 1.*** *List of the most common ID diagnoses.*

| ICD10 code | Full description | Cases [n] | Cumulative cases [n] | Cumulative cases [%] |
| --- | --- | --- | --- | --- |
| N39.0 | Urinary tract infection | 2,025 | 2,025 | 12,1% |
| J18.8 | Other pneumonia, organism unspecified | 1,152 | 3,177 | 17,8% |
| J12.8 | Other viral pneumonia | 1,083 | 4,260 | 23,8% |
| A46 | Erysipelas | 454 | 4,714 | 26,4% |
| A41.51 | Sepsis due to *Escherichia coli [E. coli]* | 394 | 5,108 | 28,6% |
| J44.09 | Chronic obstructive pulmonary disease with acute lower respiratory tract infection: FEV1 not specified | 383 | 5,491 | 30,7% |
| T84.5 | Infection and inflammatory reaction associated with a joint prosthesis | 381 | 5,872 | 32,8% |
| J36 | Peritonsillar abscess | 300 | 6,172 | 34,5% |
| J06.8 | Other acute upper respiratory infections of multiple sites | 296 | 6,468 | 36,2% |
| J44.00 | Chronic obstructive pulmonary disease with acute lower respiratory tract infection: FEV1 < 35% of the predicted value | 292 | 6,760 | 37,8% |
| L03.01 | Cellulitis of finger | 252 | 7,012 | 39,2% |
| L03.10 | Cellulitis of upper limb | 246 | 7,258 | 40,6% |
| A49.8 | Other bacterial infections of unspecified site | 230 | 7,488 | 41,9% |
| K57.32 | Diverticulitis of the large intestine without perforation, abscess, or indication of bleeding | 225 | 7,713 | 43,1% |
| B34.2 | Coronavirus infection of unspecified site | 222 | 7,935 | 44,4% |
| N45.9 | Orchitis, epididymitis, and epididymo-orchitis without abscess | 210 | 8,145 | 45,6% |
| J44.01 | Chronic obstructive pulmonary disease with acute lower respiratory tract infection: FEV1 ≥ 35% and < 50% of the predicted value | 197 | 8,342 | 46,7% |
| A41.8 | Other specified sepsis | 194 | 8,536 | 47,7% |
| A41.9 | Sepsis, unspecified | 188 | 8,724 | 48,8% |
| A41.58 | Sepsis due to other Gram-negative organisms | 184 | 8,908 | 49,8% |
| K57.22 | Diverticulitis of the large intestine with perforation and abscess, without mention of bleeding | 174 | 9,082 | 50,8% |
| J69.0 | Pneumonia caused by food or vomit | 173 | 9,255 | 51,8% |
| T84.6 | Infection and inflammatory reaction due to internal fixation device | 171 | 9,426 | 52,7% |
| J20.9 | Acute bronchitis | 165 | 9,591 | 53,6% |
| L02.4 | Skin abscess, furuncle, and carbuncle on the extremities | 151 | 9,742 | 54,5% |
| L05.0 | Pilonidal cyst with abscess | 133 | 9,875 | 55,2% |
| J22 | Acute lower respiratory tract infection | 130 | 10,005 | 56,0% |
| N45.0 | Orchitis, epididymitis, and epididymo-orchitis with abscess | 127 | 10,132 | 56,7% |
| T82.7 | Infection and inflammatory reaction due to other devices, implants, or grafts in the heart and blood vessels | 127 | 10,259 | 57,4% |
| K57.31 | Diverticulosis of the colon without perforation or abscess, with bleeding | 124 | 10,383 | 58,1% |
| J10.1 | Influenza with other respiratory manifestations, seasonal influenza viruses identified | 122 | 10,505 | 58,8% |
| A04.70 | Clostridium difficile enterocolitis without megacolon, without other organ complications | 121 | 10,626 | 59,4% |
| L02.2 | Skin abscess, furuncle, and carbuncle | 117 | 10,743 | 60,1% |

*Cases with ICD-10 codes classified as IDs were listed in descending order based on the number of cases per ICD-10 code.*

***Supplementary Table 2.*** *Proportion of mutually exclusive infection groups in the total ID population.*

| Infection groups | Cases | Age (18-49 years) | Age (50-79 years) | Age (≥ 80 years) |
| --- | --- | --- | --- | --- |
| Bones, joint, and spinal infections | 1,032 (5.8) | 236 (6.4) | 577 (6.7) | 219 (4.0) |
| CNS infections | 202 (1.1) | 88 (2.4) | 89 (1.0) | 25 (0.5) |
| Covid-19 | 312 (1.7) | 85 (2.3) | 150 (1.7) | 77 (1.4) |
| Fungal infections | 182 (1.0) | 28 (0.8) | 110 (1.3) | 44 (0.8) |
| Gastrointestinal infections | 1,636 (9.2) | 413 (11.2) | 810 (9.3) | 413 (7.5) |
| HIV-related diseases | 77 (0.4) | 52 (1.4) | 25 (0.3) | 0 (0.0) |
| Infectious heart diseases | 107 (0.6) | 12 (0.3) | 57 (0.7) | 38 (0.7) |
| Lower respiratory tract infections | 1,711 (9.6) | 142 (3.8) | 1,064 (12.3) | 505 (9.2) |
| Mycobacterial diseases | 272 (1.5) | 185 (5.0) | 76 (0.9) | 11 (0.2) |
| Other infections | 736 (4.1) | 122 (3.3) | 334 (3.9) | 280 (5.1) |
| Pneumonia | 3,535 (19.8) | 362 (9.8) | 1,694 (19.5) | 1,479 (26.9) |
| Prosthesis-, implant-, and graft-associated infections | 788 (4.4) | 88 (2.4) | 500 (5.8) | 200 (3.6) |
| Protozoan infections | 96 (0.5) | 64 (1.7) | 32 (0.4) | 0 (0.0) |
| Sepsis | 1,256 (7.0) | 62 (1.7) | 651 (7.5) | 543 (9.9) |
| Skin and soft tissue infections | 2,136 (11.9) | 901 (24.3) | 842 (9.7) | 393 (7.1) |
| Upper respiratory tract infections | 1,033 (5.8) | 484 (13.1) | 324 (3.7) | 225 (4.1) |
| Urinary tract infections | 2,767 (15.5) | 380 (10.3) | 1,339 (15.4) | 1,048 (19.1) |
| *Data are presented as numbers (percentages).* | | | | |

***Supplementary Table 3.*** *Distribution of the CMI and revenue volume of cases.*

|  | ID population | | Reference population | | Total population | |
| --- | --- | --- | --- | --- | --- | --- |
|  | CMI | Revenue volume of cases in € | CMI | Revenue volume of cases in € | CMI | Revenue volume of cases in € |
| 2020 | 1.286 | 4,697.32 | 1.068 | 3,902.87 | 1.092 | 3,988.67 |
| 2021 | 1.438 | 5,377.19 | 1.084 | 4,054.47 | 1.124 | 4,201.28 |
| 2022 | 1.395 | 5,335.84 | 1.097 | 4,196.44 | 1.129 | 4,319.92 |
| 2023 | 1.289 | 5,145.14 | 1.129 | 4,508.36 | 1.148 | 4,583.13 |
| 2024 | 1.293 | 5,432.09 | 1.157 | 4,862.95 | 1.174 | 4,933.78 |
| 2025 | 1.430 | 6,319.88 | 1.197 | 5,288.78 | 1.225 | 5,415.52 |
| Total (2020-2025) median (IQR) | 1.344 (1.290, 1.421) | 5,356.51 (5,192.82, 5,418.36) | 1.113 (1.088, 1.150) | 4,352.40 (4,089.96, 4,774.30) | 1.139 (1.125, 1.168) | 4,451.52 (4,230.94, 4,846.12) |
| Cases in ID department (2020-2025) median (IQR) | 1.281 (1.207, 1.324) | 4,955.82 (4,859.10, 5,203.05) | 1.256 (1. 090, 1.314) | 5,001.71 (4,706.09, 5,172.17) | 1.248 (1.171, 1.307) | 4,987.44 (4,724.06, 5,065.97) |
| Cases in another specialty department (2020-2025) median (IQR) | 1.389 (1.325, 1.471) | 5,468.85 (5,247.76, 5,749.84) | 1.108 (1.083, 1.149) | 4,330.55 (4,071.43, 4,769.10) | 1.128 (1.113, 1.169) | 4,411.35 (4,185.95, 4,851.95) |
| *The case-mix index (CMI) reflects the severity of the case and serves as the basis for calculating the revenue volume. The revenue per case was calculated from the CMI multiplied by the G-DRG national base rate for the Free State of Saxony [10].* | | | | | | |

***Supplementary Table 4.*** *Length of stay in the different infection groups.*

| Infection group | Length of stay (days) | Average DRG length of stay (days) | Deviation in length of stay (days) |
| --- | --- | --- | --- |
| Bones, joint, and spinal infections | 14.0 (6.0, 29.0) | 12.0 (7.0, 23.0) | 1.0 (-3.2, 10.0) |
| CNS infections | 13.0 (7.0, 17.8) | 10.0 (8.0, 10.0) | 3.0 (-2.0, 6.8) |
| Covid-19 | 5.0 (3.0, 9.2) | 5.0 (3.0, 9.0) | 0.0 (0.0, 0.0) |
| Fungal infections | 6.0 (3.0, 14.0) | 9.0 (6.0, 12.0) | -2.0 (-5.8, 4.0) |
| Gastrointestinal infections | 5.0 (2.0, 9.0) | 4.0 (4.0, 9.0) | -1.0 (-2.0, 2.0) |
| HIV-related diseases | 13.0 (7.0, 22.0) | 11.0 (8.0, 14.0) | 1.0 (-2.0, 7.0) |
| Infectious heart diseases | 18.0 (7.5, 30.0) | 26.0 (24.0, 26.0) | -4.0 (-15.5, 5.0) |
| Lower respiratory tract infections | 8.0 (5.0, 12.0) | 6.0 (6.0, 8.0) | 1.0 (-1.0, 4.0) |
| Mycobacterial diseases | 17.5 (5.0, 42.0) | 12.0 (6.0, 40.2) | 0.0 (-1.0, 0.0) |
| Pneumonia | 8.0 (5.0, 12.0) | 7.0 (7.0, 11.0) | 0.0 (-3.0, 2.0) |
| Prosthesis-, implant-, and graft-associated infections | 15.0 (7.0, 28.0) | 16.0 (7.0, 22.0) | 1.5 (-4.0, 10.0) |
| Protozoan infections | 4.0 (3.0, 5.0) | 7.0 (6.0, 9.0) | -3.0 (-4.0, -2.0) |
| Sepsis | 9.0 (6.0, 15.0) | 10.0 (9.0, 14.0) | -1.0 (-2.0, 3.0) |
| Skin and soft tissue infections | 5.0 (3.0, 9.0) | 5.0 (4.8, 7.0) | -1.0 (-2.0, 3.0) |
| Upper respiratory tract infections | 4.0 (3.0, 7.0) | 3.0 (3.0, 4.0) | 0.0 (0.0, 2.0) |
| Urinary tract infections | 7.0 (5.0, 9.0) | 6.0 (5.0, 6.0) | 1.0 (-1.0, 3.0) |
| *Data are presented as median (interquartile range [IQR]: Q1, Q3).* | | | |

***Supplementary Table 5.*** *In-hospital mortality and adjusted odds ratios for in-hospital mortality by infection group.*

| Infection group | Deaths | Cases | Percentage of deaths | OR (95% CI) | P-value | aOR (95% CI) | P-value |
| --- | --- | --- | --- | --- | --- | --- | --- |
| Bones, joint, and spinal infections | 49 | 1,032 | 4.75 | 1.49 (1.10, 1.97) | 0.007 | 1.13 (0.82, 1.52) | 0.003 |
| CNS infections | 14 | 202 | 6.93 | 2.23 (1.24, 3.70) | 0.004 | 2.61 (1.39, 4.52) | <0.001 |
| Covid-19 | 21 | 312 | 6.73 | 2.16 (1.34, 3.28) | <0.001 | 2.21 (1.35, 3.42) | <0.001 |
| Fungal infections | 12 | 182 | 6.59 | 0.56 (0.20, 1.22) | 0.2 | 1.76 (0.91, 3.11) | 0.070 |
| Gastrointestinal infections | 43 | 1,636 | 2.63 | 0.81 (0.59, 1.08) | 0.2 | 0.99 (0.99, 1.00) | 0.13 |
| HIV-related diseases | 2 | 77 | 2.60 | 0.80 (0.13, 2.53) | 0.8 | 1.01 (0.97, 1.05) | 0.8 |
| Infectious heart diseases | 15 | 107 | 14.00 | 4.88 (2.72, 8.17) | <0.001 | 2.84 (1.53, 4.93) | <0.001 |
| Lower respiratory tract infections | 144 | 1,711 | 8.42 | 2.75 (2.30, 3.26) | <0.001 | 1.50 (1.24, 1.79) | <0.001 |
| Mycobacterial diseases | 5 | 272 | 1.84 | 0.56 (0.20, 1.22) | 0.2 | 1.48 (0.52, 3.29) | 0.4 |
| Pneumonia | 580 | 3,535 | 16.4 | 5.88 (5.34, 6.45) | <0.001 | 3.15 (2.85, 3.49) | <0.001 |
| Prosthesis-, implant-, and graft-associated infections | 46 | 788 | 5.84 | 1.86 (1.36, 2.47) | <0.001 | 0.95 (0.68, 1.29) | 0.8 |
| Protozoan infections | 1 | 96 | 1.04 | 0.32 (0.02, 1.41) | 0.2 | 1.00 (0.96, 1.03) | 0.9 |
| Sepsis | 390 | 1,256 | 31.10 | 13.5 (11.9, 15.2) | <0.001 | 5.79 (5.05, 6.62) | <0.001 |
| Skin and soft tissue infections | 21 | 2,136 | 0.98 | 0.30 (0.19, 0.44) | <0.001 | 0.99 (0.98, 1.00) | 0.006 |
| Upper respiratory tract infections | 26 | 1,033 | 2.52 | 0.77 (0.51, 1.12) | 0.2 | 1.01 (1.00, 1.02) | 0.14 |
| Urinary tract infections | 42 | 2,767 | 1.52 | 0.46 (0.33, 0.62) | <0.001 | 0.98 (0.97, 0.98) | <0.001 |
| *Abbreviations: aOR, adjusted odds ratio; CI, confidence interval.* | | | | | | | |
